# Training and Experience in Study Selection (TESS): study protocol for a pilot randomised trial within a systematic review

**DOI:** 10.12688/hrbopenres.14129.1

**Published:** 2025-04-14

**Authors:** Elayne Ahern, Temitayo Adedeji, Aoife Whiston, Sarah Dillon, Fiona Lynn

**Affiliations:** 1 Department of Psychology, University of Limerick, Castletroy, Limerick, V94 T9PX, Ireland; 2 Health Research Institute, University of Limerick, Castletroy, Limerick, V94 T9PX, Ireland; 3 School of Allied Health, University of Limerick, Castletroy, Limerick, V94 T9PX, Ireland; 4 School of Nursing and Midwifery, Queen's University Belfast, Belfast, BT9 7BL, UK

**Keywords:** higher education, novice, interrater reliability, study selection, study within a review, SWAR, systematic review, training

## Abstract

**Background:**

Systematic reviews can be resource-intensive and require timely completion, yet limited availability of experienced reviewers often necessitates incorporating novice members into review teams. The purpose of this Study Within A Review (SWAR) will be to determine whether training and level of experience within the screening pair affects the reliability of decisions made by novice screeners during study selection for a systematic review

**Methods:**

A 2(training: task-specific, minimal guidance) x 2(experience level of screening partner, ‘Reviewer 1’: moderate experience, minimal experience) pilot randomised trial will be conducted within a host systematic review in the topic area of depression and psychosocial functioning. Participants ( *N* = 12), consisting of higher education students with no prior experience in evidence synthesis, will be randomised to one of the four conditions to complete a standardised study selection task at title/abstract level ( *k* = 219 records) on Covidence systematic review screening software, blindly and independently. Total participation time is estimated at 5 hours. Screening decisions made by participants will be assessed for reliability against the consensus-based decisions by two reviewers with content and methodological expertise (expert standard), through calculation of chance-corrected Cohen’s kappa and percentage of agreement, then compared across the conditions. Secondary outcomes will include reliability within the screening pair (participant and allocated screening partner), validity of screening decisions (false positives, false negatives, sensitivity, specificity), feasibility measures, including time taken to complete the study selection task and success of blinding, as well as acceptability.

**Conclusions:**

Findings will be used to inform the design of subsequent trial work to determine the efficacy of training and screener pairing for study selection. Ultimately, these insights will help to build capacity among novice screeners to engage with evidence synthesis and work alongside experienced review teams.

**Registration:**

Northern Ireland Hub for Trials Methodology Research SWAR Registry: SWAR 38.

## Background

Systematic reviews have gained prominence with a 20-fold increase in the number of systematic reviews produced from 2009–2019 ( Hoffmann *et al*., 2021). This type of review involves a rigorous synthesis of studies on a chosen topic, aiming to assess the current state of knowledge and identify gaps in the literature through a systematic approach from literature searching, through to study selection, data extraction, quality appraisal or assessment and, finally, synthesis ( Siddaway *et al*., 2019). For this reason, systematic reviews are highly regarded as a basis for evidence-based policy and practice recommendations. However, systematic reviews require time and significant human resources for completion due to their comprehensive and rigorous approach to identifying and synthesising all relevant literature in a given topic area ( Borah *et al*., 2017). This can present as a barrier to timely completion and necessitates that consideration is given towards how to improve efficiencies in the process, whilst also maintaining high quality standards.

Specifically, study selection is a resource-intensive step in the process. As per international best practice recommendations ( Edwards *et al*., 2002; Waffenschmidt *et al*., 2019), study selection should involve at least two independent reviewers to ‘screen’ study records, and thus can be referred to as ‘screeners’ for this specific stage of the systematic review ( Stoll *et al*., 2019). By accurately removing records at the earliest stages, study selection is key to efficiency in the process. Moreover, study selection is integral to ensuring that relevant studies proceed through to inclusion in the systematic review, supporting a complete and valid synthesis of evidence relevant to the review question ( Polanin *et al*., 2019). The use of two screeners offers several benefits, such as ensuring consistent application of the eligibility criteria (i.e. determining which records should be included or excluded in order to address the review question) to avoid any systematic errors as well as reducing the likelihood of any random errors by one screener compromising the accurate selection of records ( Waffenschmidt *et al*., 2019). Having at least two, independent screeners on each study record helps to ensure a transparent and reproducible method where the eligibility criteria have been applied appropriately and consistently throughout, as agreed by both screeners ( Edwards *et al*., 2002). This can be evaluated through the calculation of interrater reliability, or a chance-corrected estimate known as Cohen’s kappa ( Cohen, 1960), which is more conservative and accounts for chance, spurious agreement.

However, the recommendation of two or more reviewers to screen each study record is not always feasible. Researchers with content and methodological expertise ( Lasserson *et al*., 2021) may not be available to complete vast amounts of screening for study selection within defined project periods. Furthermore, systematic reviews are often constrained by tight deadlines and limited budgets, which can hinder their completion within the required timeframe ( Waffenschmidt *et al*., 2019). Consequently, systematic review teams may comprise of not only experienced researchers ( Lasserson *et al*., 2021) but also more novice team members such as student supervisees who undertake research as part of their programme of study (e.g., Tendal *et al*., 2009). Although rapid review methods present as a solution, allowing for a timely response to inform evidence-based decision making, these methods can be deemed responsive to situational constraints and the urgency for the evidence ( Moons *et al*., 2021). For example, only one database may be searched or study selection undertaken by just one screener. Rapid reviews, although adhering to core standards of systematic review ( Garritty *et al*., 2021; Nussbaumer-Streit *et al*., 2023), are not a substitute for the comprehensiveness and rigour of the full systematic review process. Thus, understanding how efficiencies can be achieved in the systematic review process is still of utmost interest.

Study selection methods are relatively under explored ( *k* = 11; Robson *et al*., 2019) compared to other steps in the systematic review process such as data extraction and study appraisal. Current guidance primarily focuses on the study selection procedure itself (e.g., single vs. dual screening, Gartlehner *et al*., 2020; Waffenschmidt *et al*., 2019; titles-first vs. title and abstract, Mateen *et al*., 2013), while practical guidelines on the composition of the screening team, particularly in terms of experience level, have received little attention. The few studies that address this, such as Cooper *et al*. (2006) and Ng *et al*. (2014), have found that the experience level of screeners can impact performance during study selection. Cooper and colleagues (2006), for example, examined how novice screeners, either professional dieticians or graduate nutrition students, compare with an expert standard (PhD trained nutritional scientists) during study selection for records regarding the diet-disease relationship. Findings indicated that interrater reliability within the pairs of professionals or pairs of students did not significantly differ, nonetheless, important differences between these pairs with the expert standard were observed. Novice screener pairs, whether professionals or students, may remove potentially eligible studies during title and abstract screening and thus impact the accuracy of study selection. This is further underscored by Ng *et al*. (2014) who found that study selection undertaken by medical students with no prior experience in systematic reviews varied to a large degree, with only a modest level of agreement (sensitivity) with the experienced reviewer.

Further, no studies have been identified on the role of training to improve study selection outcomes among novice screeners, although training has been assessed for other steps of the systematic review process such as quality appraisal (e.g., Acosta *et al*., 2020; da Costa *et al*., 2017; Oremus *et al*., 2012). For example, Acosta and colleagues (2020) explored the effect of a 2-hour structured training on reducing errors and bias during the quality appraisal stage of a systematic review among 14 doctoral students, with no prior experience in systematic reviews. This training included a practice activity where the novices compared their scores across the nine criteria on the Methodological Quality Questionnaire for a given study record to that of the expert, discussing hits (agreements) and misses (disagreements) for the practice activity. The student reviewers were randomly assigned one of three study records of varying quality for appraisal. Following independent completion of the study quality appraisal task, and when compared to expert raters, 43% of the novice student reviewers accurately assessed 7 out of 9 (78%) methodological quality criteria when evaluating the quality of the study record. Training-based benefits have not always been observed among reviewers with already established expertise ( Fourcade *et al*., 2007) or even among novice student reviewers ( Oremus *et al*., 2012) during the quality assessment phase. Nonetheless, when formats of training (e.g., intensive vs. minimal) have been investigated among novice student reviewers, findings have suggested that the content of training could influence the likelihood of positive training effects. da Costa *et al*. (2017) examined minimal training, consisting of an introductory 1-hour lecture on risk of bias assessment, with intensive training, which included the same lecture but with specific assessment instructions provided by an experienced reviewer (vs. reading material provided to the minimal training condition). Further, the student reviewers who received intensive training then undertook a practice assessment in a purposely selected sample of 10 records, with assessment decisions then discussed by the experienced reviewer. Overall, this produced more favourable kappa estimates for both within- and between-group interrater reliability, as novice screeners who received the intensive training were afforded an opportunity to calibrate their assessments to that of the experienced reviewer during the practice task. This underscores the value of specific training in enhancing the ability of novice reviewers to engage with systematic review processes.

Alongside the role of training, there is reason to believe that the pairing of novice screeners with more experienced review team members when undertaking study selection could help improve reliability estimates. As evidenced by da Costa *et al*. (2017), interrater reliability estimates can be improved when the opportunity is afforded to novice reviewers to align their decisions with that of the experienced reviewer. During study selection, the use of software such as Covidence (Veritas Health Innovation) can help to streamline the process among screeners and promptly notify of conflict (disagreement) detection between screeners, offering an opportunity for novice screeners to subsequently adjust and re-calibrate their performance. As such, the experience level of the screening partner is also of interest. To the best of our knowledge, studies that have examined novice reviewer performance in dual assessment tasks have only ever done so investigating novice-novice pairings (e.g., da Costa *et al*., 2017; Oremus *et al*., 2012). Although experience level has not always been associated with more valid and reliable performance during systematic review processes (e.g. Horton *et al*., 2010; data extraction), it is plausible that novice reviewers could use conflict detection to recognise patterns of disagreements during study selection as a result of exposure to more expert decision-making and adjust their approach for subsequent study records accordingly. As per guidelines, explicit discussions should be had within the review team to resolve conflicts and consolidate the rationale behind the decision to include or exclude a study record ( Lefebvre *et al*., 2024). Nonetheless, the active pairing of a novice reviewer with a more experienced reviewer could offer a referential standard through real-time feedback during the screening process, helping to overall improve efficiency. Real-time feedback on conflict detection during dual screening with an experienced reviewer could help to consolidate the impact of training, however, this has not yet been investigated.

### Aim and objectives

Systematic reviews are considered the highest form of evidence, guiding policy and practice through the transparent and rigorous synthesis of research ( Haddaway & Pullin, 2014). The reliability of study selection is integral to this process, particularly when review teams include more novice screeners. Inconsistent selection processes can directly influence which studies are included, ultimately shaping the available evidence base upon which recommendations are developed. Thus, the aim of this pilot study is to determine whether training and level of experience within the screening pair affects the reliability of decisions made by novice screeners during study selection for a systematic review. Drawing on the Study Within A Review (SWAR; Devane *et al*., 2022) methodology, where a study is embedded within a systematic review in order to address questions of methodological uncertainty during the review process, we propose the following objectives:

(i) To examine whether experience level of the screening partner and the provision of training affects the reliability of screening decisions made by novice, student screeners (between-group reliability).
(ii) To examine whether pairing a novice, student screener with an experienced screener (vs. novice screener), alongside the provision of task-specific training (vs. minimal guidance), improves reliability estimates within the screening pair during the study selection process (within-group reliability).
(iii) To explore the feasibility and acceptability of training materials for study selection by novice, student screeners.

Specifically, we hypothesise that reliability in study selection will be highest among the group of novice screeners that receive task-specific training and are paired with an experienced screener.

## Methods

This pilot study will be conducted in accordance with SWAR guidance ( Devane *et al*., 2022) and is pre-registered on the Northern Ireland Network for Trials Methodology Research SWAR Repository Store (SWAR 38; see ‘Extended Data’ section below, Ahern *et al*., 2025), with notice of acceptance on 4 February 2025. The protocol is reported according to SPIRIT guidelines for trial protocols (Standard Protocol Items: Recommendations for Interventional Trials; Chan *et al*., 2013), and supplemented by the CONSORT extension to pilot trials ( Eldridge *et al*., 2016), as is recommended for the reporting of pilot and feasibility study protocols ( Thabane & Lancaster, 2019). See the completed SPIRIT and CONSORT extension checklists, available from the OSF link provided in the ‘Reporting Guidelines’ section below. Any modifications to the protocol will be recorded and subsequently presented in the pilot study report.

### Study design

**Figure 1.**
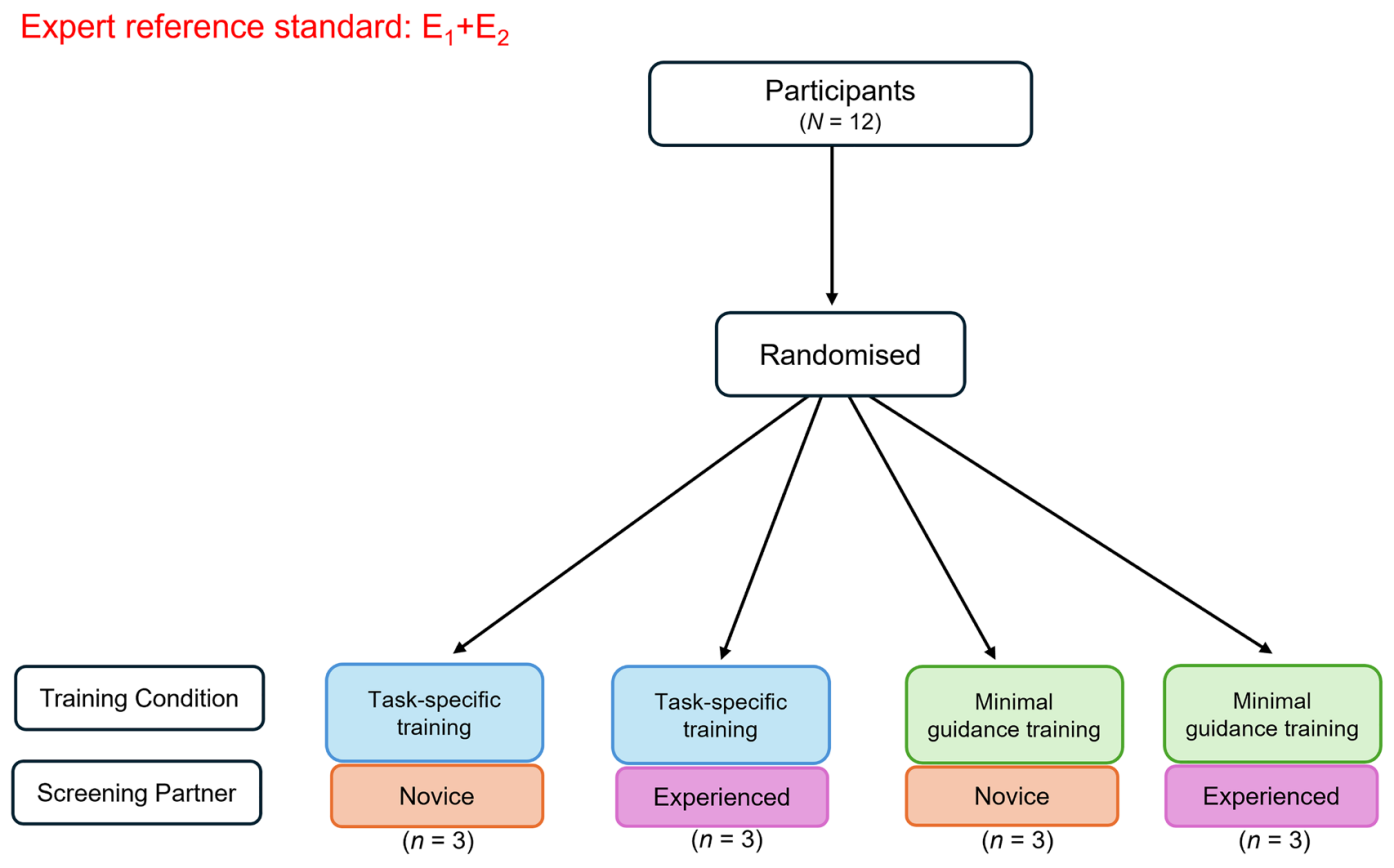
Flow diagram of randomisation to the respective pilot study conditions. *Figure Note*. Participants ( *N* =12) will be randomised to a training condition (task-specific or minimal guidance) and screening partner (novice screener or experienced screener). The consensus-based decisions of the expert reviewers (E1, E2) will inform the expert reference standard. E1 = Expert reviewer 1 with content and methodological expertise; E2 = Expert reviewer 2 with content and methodological expertise.

This SWAR is a pilot study that will employ a 2x2 factorial randomised controlled trial (RCT) design. Participants will be randomised using a random number generator for simple randomisation to one of four conditions: (i) task-specific training with a novice screening partner, (ii) task-specific training with an experienced screening partner, (iii) minimal guidance training with a novice screening partner, and (iv) minimal guidance training with an experienced screening partner (see Figure 1). A member of the research team (AW) has computer-generated the randomisation sequence (conditions A, B, C, D) in advance of recruitment and will be kept blind to treatment allocation as well as data analysis. Recruitment and onboarding of participants will be separately managed by an unblinded research assistant coordinator (TA), who following participant informed consent, will contact the respective team member that handles randomisation to determine the next pre-determined allocation in the sequence. Members of the team that will be involved in data analysis (EA) will remain blinded to allocation to ensure minimal risk of bias to data analysis. Participants will remain blinded to their allocated group throughout the study. Ethical approval for this study has been obtained from the Faculty of Education and Health Sciences Research Ethics Committee at the University of Limerick (EHS Approval Number: 2024_06_24_EHS).

### Study selection task

**Study records.** An on-going systematic review project led by the PI (PROSPERO registration: CRD42022324367) will be used to host the SWAR. This host review addresses non-pharmacological treatments for psychosocial functioning in major depressive disorder. Please see ‘Extended Data’ ( Ahern *et al*., 2025) for a sample database search strategy. A comprehensive search of the literature has been undertaken across 6 databases, identifying in excess of 12,500 potentially eligible study records from inception through to the most recent updated search in August 2024. From this, a sample of 2% ( *k* = 219) has been identified for use in the study selection task for this SWAR. This sample has been selected by the PI to represent the full breadth of eligibility criteria so as to best capture the efficacy of training to promote high reliability during title/abstract study selection. Based on calculations using the kappaSize package ( Rotondi, 2018) and PowerBinary function on r, 219 records were determined for the study selection task in order to achieve 80% power to detect a significant difference in reliability assuming kappa = 0.51 (weak/fair agreement, McHugh, 2012; Orwin, 1994) between the expert standard and the minimal guidance training+novice screening partner condition relative to kappa = 0.71 (moderate/good agreement, McHugh, 2012; Orwin, 1994) between the expert standard and task-specific training+experienced screening partner condition, with an alpha level of .05, and assuming a 20% prevalence of include at title/abstract level. Consistent with other SWAR methodologies assessing rater agreement ( Cooper *et al*., 2006; da Costa *et al*., 2017), power calculations are used to determine the adequate number of study records that need to be assessed to ensure the calculation of reliable kappa statistics. Prior to finalising the selection of records to be used for the screening task, a pilot sample of *k* = 20 records have been selected for screening within the review team (EA, AW), with the rationale for inclusion/exclusion documented. Interrater reliability, that is both reviewers agreeing ‘YES’ to include or ‘NO’ to exclude to each respective study record, was 100% (κ = 1.00) suggesting perfect agreement ( Cohen, 1960; McHugh, 2012). The reference list of study records for the study selection task are available on the project OSF, see ‘Extended Data’ ( Ahern *et al*., 2025).

**Expert standard.** All study records have been screened independently by two members of the review team (EA, AW) with several years of methodological experience in evidence synthesis and content expertise in the area of depression ( *M* = 9.5, *SD* = 1.41 years of experience). As per the criteria proposed by Horton *et al*. (2010), EA and AW would meet the standards of ‘substantial experience’ in systematic review (>7 years involved in systematic reviews, >7 reviews for which involved in at least study selection). Screening has been completed on separate Covidence project sites to ensure complete independence in decision making. Any screening discrepancies have been resolved through discussion with the consensus-based decisions then used as the expert standard. The interrater reliability was 93.15% agreement (κ = 0.77, SE = 0.06, 95% CI[0.66, 0.88]).

**Screening partner (‘Reviewer 1’).** A novice member of the review team (TA) with ‘minimal experience’ (≤2 years involved in systematic reviews, ≤2 systematic reviews for which involved in at least study selection) and another member (SD) with ‘moderate experience’ in systematic review methodology (4 – 6 years involved in systematic reviews, 4 – 6 systematic reviews for which involved in at least study selection), as per the classifications proposed by Horton *et al*. (2010), have independently completed screening for the selected records on separate Covidence project sites to avoid influencing their respective screening decisions. The screening decisions made by TA and SD will then be used for the ‘Screening Partner’ condition, corresponding to novice and experienced screening partner, respectively. The screening decisions have been loaded on to Covidence project sites in advance and described as the screening decisions made by ‘Reviewer 1’. Participants will be randomly allocated to one of two screening partners in combination with a training condition (task-specific, minimal guidance; See ‘Training’ section below).

### Participants

A total of 12 participants (N = 12) will be recruited within an anticipated 3-month timeframe. Recruitment commenced in February 2025. As this SWAR is designed as a pilot study, power analyses were not used to determine sample size and it was deemed appropriate to recruit a small number of participants per trial condition, consistent with other SWAR methodologies (e.g., Acosta *et al*., 2020; Cooper *et al*., 2006; da Costa *et al*., 2017; McGuire *et al*., 1985; Oremus *et al*., 2012). Further, the small sample size is appropriated given that this pilot trial is not intended for hypothesis-testing but more so for exploration of hypotheses, and to assess the feasibility and acceptability of the proposed methods to inform the design of a subsequent fully powered trial.

Participants will be recruited from higher education institutions with convenience sampling of health sciences students from the University of Limerick in the first instance given the small target sample size and the access afforded to the research team given roles in teaching at undergraduate and postgraduate level within the discipline. Information regarding the study will be disseminated through course site postings on the virtual learning environment, with word-of-mouth and snowball sampling methods also employed. The eligibility criteria for participation include: (i) being at least 18 years of age; (ii) being enrolled as a student in higher education; (iii) having no prior training or experience in conducting evidence synthesis; and (iv) having access to a laptop or computer with a reliable internet connection. Participants will be entered into a draw to win a monetary prize (2 x €50 vouchers) as renumeration for their participation.

### Training conditions

Both the task-specific training and minimal guidance training conditions will receive a standardised outline of eligibility criteria for study selection (see ‘Extended Data’, Ahern *et al*., 2025). Additionally, a recorded online training with narration, embedded video content, and slide deck will be provided, but the extent of training will differ between the conditions (task-specific vs. minimal guidance). Covidence Support (Veritas Health Innovation) provides a bank of online training videos which have served as a point of reference in developing the content for this session and instructional guide on use of the software.

The content of the training has been piloted within the review team before finalisation. Both the training and control have been time-matched for an estimated completion time of 45 minutes, consisting of the following core content: an introduction to the review topic area, what is a systematic review, study selection and eligibility criteria, an introduction to Covidence systematic review software, guidelines for study selection, and instructions for the study selection task.

**Control (minimal guidance training).** In addition to the core training content, the control comparator training session includes extended content on what is a systematic review, considering when to or when not to conduct a systematic review and instruction on some reporting guidelines, such as the PRISMA flow diagram to visualise the study selection process. Specifically, the content relevant to study selection and Covidence is generalised and not specific to the host review.

**Intervention (task-specific training).** In addition to the core training content, an extended discussion on eligibility criteria for the host review is provided, offering clarification and recommendations when screening, followed by a demonstration of screening for a sample of 10 records in the review topic area, not included in the sample of records for the study selection task, with clear rationale provided for the screening decisions made. Similar online training approaches with a sample demonstration of records, reflecting what is likely to be encountered during screening, have been implemented for novice screeners (‘crowd,’ non-specialist) on systematic review projects ( Noel-Storr *et al*., 2021). The purpose of this training is to refine knowledge and understanding specific to engaging with study selection for the host review.

For ease of access, all details will be hosted on a virtual learning environment project site, separate for each training condition. A sample of the project site interface is available from the project OSF (see ‘Extended Data’, Ahern *et al*., 2025). Across both the task-specific and minimal guidance conditions, participants will start at the first unit, ‘TESS Study: Introduction’, followed by ‘The Research Team’, and then proceed through Steps 1–5 accordingly: (1) ‘Demographic and Background Questionnaire’, (2) ‘Training Materials’, (3) ‘Access the Study Selection Task’, (4) ‘The Study Selection Task’, (5) ‘Post-Task Questionnaire’.

### Measures and outcomes

**Interrater reliability.** All relevant performance outcome data will be recorded on Covidence systematic review screening software. Participants will be instructed to select between two options, ‘YES’ (include) and ‘NO’ (exclude). This will allow for the calculation of an unweighted, chance-corrected Cohen’s kappa ( Cohen, 1960) as the response decisions are dichotomous. Based on the criteria set out by McHugh (2012), Cohen’s kappa values can be interpreted as no agreement (κ = ≤ 0.20), minimal (κ = 0.21 - 0.39), weak (κ = 0.40 - 0.59), moderate (κ = 0.60 - 0.79), strong (κ = 0.80 - 0.90), or almost perfect (κ > 0.90). Furthermore, the percentage agreement will be reported alongside Cohen’s (1960) kappa, as per recommendations ( McHugh, 2012) given that the kappa statistic can underestimate reliability due to the underlying assumptions made on chance rater agreement.

Primary and secondary outcomes are:

Primary (i) between-group reliability, defined as the reliability between the decisions of the participants and the expert standard, calculated as percentage agreement and unweighted, chance-corrected Cohen’s kappa ( Cohen, 1960). The mean kappa estimate, standard error, and 95% CI will be generated for each of the four trial conditions (e.g., task-specific training and experienced screening partner; minimal guidance and novice screening partner) to enable comparison across the conditions. Between-group reliability will be calculated as, κ _ns, expert standard_ = (P _o1_ – P _e1_) / (1 - P _e1_), where P _o1_ is the proportion of records screened when the decision by the participant novice screener is in agreement with the expert standard, and P _e1_ is the proportion of records where the decision by the participant novice screener is in agreement with the expert standard by chance.

Secondary (i) within-group reliability, defined as the reliability within each pair of participant and screening partner, calculated as κ _ns, screening partner_ = (P _o2_ – P _e2_) / (1 - P _e2_), where P _o2_ is the proportion of records screened when the decision by the participant novice screener is in agreement with their allocated screening partner, and P _e2_ is the proportion of records where the decision by the participant novice screener is in agreement by chance.

Similarly, the mean kappa estimate, standard error, and 95% CI will be generated to allow for comparison across conditions, and, (ii) the validity of screening decisions made by participants relative to the expert standard, including the total number of false positives (vote ‘YES’ by participant to include irrelevant articles), false negatives (vote ‘NO’ by participant to exclude relevant articles), sensitivity (ability of participant to vote ‘YES’ to include relevant articles), and specificity (ability of participant to vote ‘NO’ to exclude irrelevant articles).

Several measures will be collected from participants through use of the online survey software tool, Qualtrics.

**Sociodemographic, education, and experience level.** A Demographic and Background Questionnaire will be administered at baseline to collect data relevant to experience in evidence synthesis, level of education, area of study, age, gender.

**Feasibility and Acceptability Outcomes.** Following screening completion, participants will again be asked to complete a brief Qualtrics questionnaire to collect data on our primary feasibility and acceptability outcomes, namely:

***Efficiency*.** Participant's self-report of the time taken in hours and minutes to complete the study selection task.

***Success of blinding*.** Participants will be asked to indicate what training condition they believe they were allocated to in order to determine the success of participant blinding procedures.

***Perceived usefulness of training and screening partner decisions in study selection*.** The perceived usefulness of the training and pairing with the screening partner in the completion of study selection will be assessed using 7 items on a Likert scale from 1 ( *strongly disagree*) to 7 ( *strongly agree*). Items include, for example, ‘ *The screening decisions of ‘Screener 1’ (whether there was a conflict in decision or not) helped me to adjust my approach to the study selection process*’ and ‘ *The training was useful in assisting me to complete study selection*’.

Secondary feasibility measures include:

***Motivation/interest*.** In response to the question ‘ *What was your main interest or motivation in participating in this study?*’, participants will be asked to rank the options in order of their main motivation and/or interest, including: interest in the topic area, interest in systematic review methods, the chance to get involved in research, skill development/capacity building in systematic review methods, and other (please specify).

***Recommendations for improvement*.** Participants will be provided with an open-ended response type question on ‘ *What, if anything, would have improved the overall experience*?’

### Procedure

**Figure 2.**
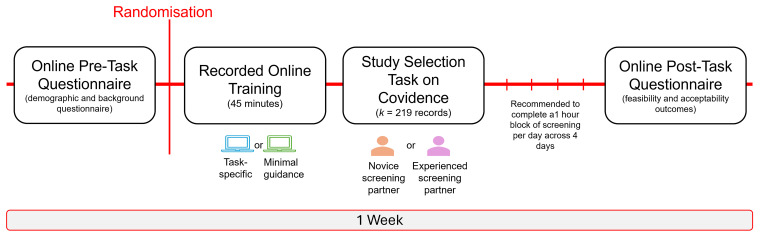
Schematic figure of participation tasks and anticipated timeline. *Figure Note*. In the instance that the study selection task has not been completed following 1-week of enrolment, a gentle e-mail reminder will be sent and then again every week for up to 3 weeks.

Individuals who express interest in participation will be provided with an information sheet and access to an online Qualtrics survey by the study research assistant (TA) where participants can record their provision of informed consent (see ‘Extended Data’, Ahern *et al*., 2025). Consenting participants will then be enrolled in the study following self-declaration that they meet the eligibility criteria. A unique identification number will be assigned to allow for participant questionnaire responses and reliability data from the study selection task on Covidence to be linked. The study research assistant (TA) will generate a pseudonymisation key, only accessible to them during the study, and this will be stored securely and separately on the institution’s cloud-based storage. Participants will proceed to complete a brief demographic and background questionnaire on Qualtrics. Participants will then be randomised to a trial condition (for randomisation process, see ‘Study Design’) and provided with instructions on how to access the training site on the virtual learning environment and their Covidence account, which has been set up on their behalf by the study research assistant (TA) through an institutional licence. Participants will be able to select their own log-in password, with their e-mail as the username. Covidence will be set to 'dual screener' mode, meaning that each study record requires a decision by two independent screeners. For the purposes of this study, only the ‘YES’ and ‘NO’ screening options will be used to avoid difficulties in ascertaining the interrater reliability of responses assessed as ‘MAYBE’. The screening decisions of ‘Reviewer 1’ will be loaded up on the respective review page on Covidence, prior to the participant commencing (as 'Reviewer 2'). Please refer to section ‘Screening Partner (‘Reviewer 1’)’ for further detail. For each record screened, the participant will be notified by Covidence as to whether they are in agreement or in conflict with ‘Reviewer 1’. The participant need only complete the study selection task at the title/abstract level, with no requirement to proceed to conflict resolution or full-text screening. Instructions on the study selection task will be provided in the training material (see ‘Extended Data’, Ahern *et al*., 2025). In brief, participants will be instructed to read the title and abstract of each study record and make a decision based on the eligibility criteria using their best judgement. If there is reason to believe that a study may be eligible, but cannot confirm at the title/abstract stage, then a conservative decision should be made to retain the study record (‘YES’ vote). If the decision conflicts with Reviewer 1’s, this is a typical part of the study selection process. No further action is needed in resolving the conflicts and this will be managed by the review team. In order to maintain the integrity of the independent screening process (between Reviewer 1 and Reviewer 2), a decision should not be changed once made. Participants will also be asked not to discuss their training with others to minimise the potential risk of contamination across the trial conditions. It will be recommended to complete the study selection task in focused, distraction free blocks (e.g., 30 minutes) to avoid screening fatigue. In total, the recommendation is to complete 1-hour of screening each day, across 4 days. Queries or any technical difficulties can be raised with the study team, however, queries directly related to the study selection task cannot be answered to avoid potentially influencing the participant’s screening decision. Upon completion of the study selection task, the participant will be invited to complete a brief questionnaire on Qualtrics to self-report on feasibility outcomes such as the time taken to complete the study selection task, success of blinding, and acceptability outcomes, including perceived usefulness of the training and screening decisions of their screening partner, motivation/interest to engage in the task, and recommendations, before being debriefed on the study. A gentle e-mail reminder will be sent following 1-week of enrolment, and then every week for up to 3 weeks in the instance that the participant has not yet completed the study selection task. Total participation time across the study conditions is estimated at 5 hours. A schematic summary of the participation timeline is provided in Figure 2.

### Analysis

Data will be collated from the Qualtrics survey and the respective project sites on Covidence systematic review screening software and then reviewed by EA for completeness and accuracy. Analysis will be conducted by EA who will remain blinded to participant allocation. Any queries related to interrater reliability will be directed to the study research assistant (TA) who will confirm the accuracy of the data from the respective Covidence project sites, ensuring that the integrity of blinding is upheld. The final dataset will be accessible via cloud-based sharing to members of the research team only.

As this SWAR is designed as a pilot study, the focus is on descriptive statistics and estimation, not hypothesis-testing ( Lee *et al*., 2014; Leon *et al*., 2011). Inferential statistics will be generated for the purposes of inferring if differences exist between the trial conditions, but conclusions on the efficacy of training or screening partner will not be drawn.

Cohen’s kappa will be calculated on r using the *irr* package ( Gamer *et al*., 2019). A mean kappa will be computed for each trial condition, along with the 95% CI and standard error. Percentage agreement in screening decisions will also be calculated as a simple percentage and interpreted alongside the respective kappa statistics. All available screening data, including partial data (i.e., screening decisions available for 1 ≤ *k* < 219 records) in the instance of study selection task non-completion, will be used in the calculation of interrater reliability. As interrater reliability can be calculated from the completion of just *k* = 1 study record, a minimum threshold of 10% record completion for the study selection task ( *k* = 22 records) is imposed for data to be included in the condition-level summaries (Mean, 95% CI) and comparison between conditions. The minimum 10% threshold is consistent with the Agency for Healthcare Research and Quality Methods guidance on the pilot phase of study selection to reduce errors and as a form of calibration in the interpretation of eligibility criteria between two independent reviewers, before a single reviewer subsequently proceeds to complete study selection ( McDonagh *et al*., 2013). This criterion ensures that reported summary statistics and comparisons will be based on a meaningful amount of data while still allowing (partially) available data from this SWAR pilot study to be appropriately incorporated. To assess the potential impact of missing data, sensitivity analyses will be conducted using only complete cases (i.e., screening decisions available for *k* = 219 records) to determine the robustness of findings.

To compare differences between the kappa estimates produced by paritipcnats (vs. expert standard) in each trial condition and within the screening pairs, bootstrapping will be conducted using the *boot* package ( Canty & Ripley, 2024; Davison & Hinkley, 1997) to generate bootstrapped CIs and *p*-values. Differences in kappa estimates will be visually presented using a forest plot. In instances where the plotted kappa estimate and 95% bootstrapped CI cross 0, it can be inferred that the respective trial conditions produce equivalent kappa estimates.

Measures of validity, including false positives, sensitivity, and specificity, will be calculated and presented in tabular form. Rank-based responses on motivation/interest to engage in the study and categorical responses indicating the success of blinding will be reported as frequencies and percentage, with open-text responses narratively outlined. Furthermore, feasibility outcomes will be presented descriptively with inferential statistics generated. For Likert scale response outcomes on acceptability, total scores will be computed and reported using *M* and *SD* estimates, with a factorial between-group ANOVA generated, where appropriate, to explore potential differences in acceptability between the groups. Efficiency in the study selection task (time taken) will be assessed descriptively and inferentially to determine if time taken for completion likely varies across the trial conditions.

## Conclusions

It is intended that the findings will be disseminated in a peer-reviewed publication and knowledge exchange event, with associated materials from the design and reporting of the project made available on the OSF project site. Findings are anticipated to provide insights into further efficiencies that can be achieved in the systematic review process by determining whether novice screeners can achieve a high standard of reliability during study selection following training and pairing with a screening partner. Thus, novice screeners could successfully be integrated into review teams to assist with project completion. Alongside this is the impact of these potential findings on capacity building for evidence synthesis among student or early career researchers. It is intended that findings will be applied to help inform the development of training for student researchers which could potentially lead to a sustainable research culture, where students have the opportunity to work alongside experienced faculty on evidence synthesis projects. To this end, the findings of this SWAR pilot trial will be used to inform the design of subsequent trial work to appropriately test the efficacy of training and screener pairings for study selection.

## Study status

At the time of protocol submission, recruitment had commenced with data collection on-going.

## Data Availability

No data are associated with this article. Open Science Framework: Training and Experience in Study Selection - The TESS Study. https://doi.org/10.17605/OSF.IO/WKHQJ. ( Ahern *et al*., 2025). This project contains the following extended data: Participant Information Sheet and Consent Form, including TESS Study Ethical Consent Form.pdf and TESS Study Participant Information Sheet.pdf Project Training Site, including TESS Study_VLE project site interface_sample.pdf Protocol Reporting Checklists, including CONSORT Extension Pilot and Feasibility Trials Checklist.pdf and SPIRIT Checklist.pdf Study Selection Task, including TESS Study Eligibility Criteria.pdf, TESS Study Eligibility Criteria_Supporting Materials.pdf, TESS Study Instructions for Study Selection Task.pdf, TESS Study Sample Database Search Strategy.pdf, and TESS Study Selection Task References.pdf Data are available under the terms of the Creative Commons Zero "No rights reserved" data waiver (CC0 1.0 Public domain dedication). The Northern Ireland Network for Trials Methodology Research. SWAR Repository Store. SWAR registration for ‘Training and Experience in Study Selection (TESS): A Pilot Randomised Trial within a Systematic Review’. SWAR 38.
