# Supplementary material for: Training and Experience in Study Selection (TESS): study protocol for a pilot randomised trial within a systematic review
Source: HRB Open Res. 2026 Apr 16;8:54. Originally published 2025 Apr 14. [Version 3] doi: 10.12688/hrbopenres.14129.3 (PMC12982981; doi:10.12688/hrbopenres.14129.3)
Supplement: Peer review history

# Reviewer report: Reviewer response for version 2

**DOI:** 10.21956/hrbopenres.15764.r53773

**Published:** 2026-03-12

**Authors:** Heather MacDonald

**Affiliations:** 1 MacOdrum Library, Carleton University, Ottawa, On, Canada

This protocol is well designed to address the study questions posed. There is currently a lack of evidence on novice screener training, and this protocol helps fill this gap.

 The changes from the first round of revisions to this protocol increases its rigour and clarity.

 I only have one comment about the open-ended question about recommendations for improvement.  There is no analysis mentioned for this.  What do you hope to gain from including this question?  With such a small sample size a thematic analysis is not appropriate.  Will you highlight any quotes? Consider adding a sentence to address this.

Is the study design appropriate for the research question?

Yes

Is the rationale for, and objectives of, the study clearly described?

Yes

Are sufficient details of the methods provided to allow replication by others?

Yes

Are the datasets clearly presented in a useable and accessible format?

Not applicable

Reviewer Expertise:

information literacy, evidence synthesis

I confirm that I have read this submission and believe that I have an appropriate level of expertise to confirm that it is of an acceptable scientific standard.

# Response

**Published:** 2026-03-31

**Authors:** Elayne Ahern

**Affiliations:** Psychology, University of Limerick Faculty of Education and Health Sciences, Limerick, Limerick, Ireland

Thank you kindly for the time and attention given to reviewing our protocol. The feedback is very much appreciated and has helped to enhance the quality of our work. For ease of reference, we have listed the peer-review comments in bold, followed immediately by our responses, presented point-by-point.

**This protocol is well designed to address the study questions posed. There is currently a lack of evidence on novice screener training, and this protocol helps fill this gap. The changes from the first round of revisions to this protocol increases its rigour and clarity.**

 Thank you for positively receiving our work and for noting the improved rigour and clarity as a result of the revisions already incorporated.

**I only have one comment about the open-ended question about recommendations for improvement. There is no analysis mentioned for this. What do you hope to gain from including this question? With such a small sample size a thematic analysis is not appropriate. Will you highlight any quotes? Consider adding a sentence to address this.**

 Thank you for this helpful suggestion as we acknowledge that we lacked sufficient detail on this in our previous manuscript version. We have now clarified the handling of the open-ended responses about recommendations, which we intend to use for the purposes of refining the intervention or study design in subsequent work. Given the small sample size and the expectation that responses will be brief, rather than in-depth, we do not plan to conduct formal qualitative analysis. Instead, the responses will be summarised descriptively, with illustrative quotes included where they help to highlight aspects of participants’ experiences and suggested improvements for future trial work.

# Reviewer report: Reviewer response for version 1

**DOI:** 10.21956/hrbopenres.15528.r50011

**Published:** 2025-09-30

**Authors:** Declan Devane

**Affiliations:** 1 University of Galway, Galway, Ireland

This protocol addresses a practical and under‑studied step of evidence synthesis: how to train and pair novice screeners so that study selection remains reliable and efficient. The rationale is well supported by prior work on dual screening, training effects, and kappa‑based reliability. Strengths include a clear factorial design, preregistration, transparency about materials, and attention to feasibility and acceptability. Overall, the protocol is clear and useful, but several aspects would benefit from clarification or modest redesign to strengthen internal validity and the interpretability of results.

 Your paper already says this is a pilot that will inform a larger trial. To make that unmistakable, add a brief sentence near the start of Methods and again in Analysis that the goal is learning and planning. Then list two or three simple progression rules, for example completion rate, time to finish, and acceptability thresholds, based on the feasibility data you already collect on pages 7 to 9.

 The current aim reads like a test of which arm is best. A small rewording might fix that e.g., “This pilot will assess feasibility and acceptability to plan a definitive trial” and align outcomes accordingly.

 Readers will appreciate a brief expansion of how the 219 records will be chosen.

 Because Covidence shows agreement or conflict as people work, learning during the task is very plausible. Suggest that the record order be randomised for each participant if possible, and report performance by time or by item deciles to show any learning pattern.

 With only three participants per cell, the averages of kappa by arm will be wide regardless. You might consider keeping those and adding a complementary item-level mixed model that treats each screened record as one observation and includes effects for training, partner type, and their interaction, with random effects for participant and item.

 In Analysis on page 9 you plan to treat confidence intervals that cross zero as evidence of equivalence. That can be misread. Suggest you avoid equivalence language or set a small, prespecified equivalence margin for kappa differences and say any tests are exploratory.

 Kappa can be sensitive to prevalence. Keep kappa and percent agreement, then add one robust agreement index such as PABAK. This may make the results easier to read if the include rate is low or high.

 You define the partner with 4 to 6 years as moderate experience on page 6, but label the arm “experienced”. Maybe revised labelling.

 Because one person represents each partner type, partner identity is confounded with partner type. Flag this as a pilot limitation and say that the full trial will include multiple partners per level.

 The Procedure seems to ask participants to vote YES when unsure. Suggest adding a sentence that explains the consequence of this rule for sensitivity and specificity, so readers know you expect higher sensitivity and possibly lower specificity by design.

 Time is self-reported in your feasibility outcomes. If Covidence logs can provide start or finish times, say that you will also capture those as an objective check on efficiency.

Is the study design appropriate for the research question?

Yes

Is the rationale for, and objectives of, the study clearly described?

Yes

Are sufficient details of the methods provided to allow replication by others?

Yes

Are the datasets clearly presented in a useable and accessible format?

Not applicable

Reviewer Expertise:

Clincial trials and evidence synthesis methodology

I confirm that I have read this submission and believe that I have an appropriate level of expertise to confirm that it is of an acceptable scientific standard.

# Response

**Published:** 2025-12-17

**Authors:** Elayne Ahern

**Affiliations:** Psychology, University of Limerick Faculty of Education and Health Sciences, Limerick, Limerick, Ireland

Thank you kindly for the time and consideration given to reviewing our protocol. For ease, we have provided the peer-review comments, formatted in bold, with our response then presented immediately thereafter, point-by-point.

**This protocol addresses a practical and under‑studied step of evidence synthesis: how to train and pair novice screeners so that study selection remains reliable and efficient. The rationale is well supported by prior work on dual screening, training effects, and kappa‑based reliability. Strengths include a clear factorial design, preregistration, transparency about materials, and attention to feasibility and acceptability. Overall, the protocol is clear and useful, but several aspects would benefit from clarification or modest redesign to strengthen internal validity and the interpretability of results.**

 Thank you for positively receiving our work. The recommendations are much appreciated, with the validity and clarity of our protocol strengthened following these suggested revisions.

**Your paper already says this is a pilot that will inform a larger trial. To make that unmistakable, add a brief sentence near the start of Methods and again in Analysis that the goal is learning and planning. Then list two or three simple progression rules, for example completion rate, time to finish, and acceptability thresholds, based on the feasibility data you already collect on pages 7 to 9.**

 Thank you for this suggestion. We have added a statement at the beginning of the Methods and again throughout the Analysis section to clarify that the primary purpose of this SWAR is to learn from pilot study findings and plan for a future definitive trial. In addition, we have now added a ‘Progression criteria’ subsection to the Methods, where we have specified criteria to guide decisions about progression and refinement of the full-scale trial based on feasibility and acceptability outcomes, including completion rate, time required of participants, and perceived usefulness of training and screener pairings when undertaking the study selection task.

**The current aim reads like a test of which arm is best. A small rewording might fix that e.g., “This pilot will assess feasibility and acceptability to plan a definitive trial” and align outcomes accordingly.**

 Thank you for this helpful suggestion. We have revised the aim to more clearly reflect the pilot purpose of assessing feasibility and acceptability to inform a future definitive trial. We have also updated the objectives and language so that reliability outcomes are framed as exploratory estimates intended to guide the design of the future trial, rather than as formal comparative tests.

**Readers will appreciate a brief expansion of how the 219 records will be chosen.**

 We have expanded the Methods section to clarify the process used to select the 219 records. Specifically, we now explain that the sample was purposefully drawn to reflect the breadth of eligibility criteria in the host review, including a proportion of records previously screened as “include” ( *k* = 44) and a diverse set of “exclude” records ( *k* = 175) to represent the full range of exclusion reasons. This ensures that participants encounter a variety of screening decisions and the need to apply various exclusion rationales, supporting a meaningful assessment of how well the training conditions prepare novice screeners to apply the eligibility criteria.

**Because Covidence shows agreement or conflict as people work, learning during the task is very plausible. Suggest that the record order be randomised for each participant if possible, and report performance by time or by item deciles to show any learning pattern.**

 Thank you for this helpful guidance. We explored options for randomising record order and analysing learning patterns. However, as confirmed by Covidence Support, Covidence software does not currently support randomisation of the screening order. Records are displayed by default using the “most relevant” ordering, and this is how the study selection task was presented to participants. The “most relevant” ordering is underpinned by Covidence’s internal active learning algorithm, although users can switch to alternative static orderings (e.g., Title or Author) if they choose. Further, the Covidence platform does not provide time-stamped screening data (only the date the record was screened is available), which prevents analyses by time or by item deciles. We recognise that not being able to explore learning during study selection is a limitation and unfortunately cannot action this for our pilot SWAR given the constraints of the platform.

**With only three participants per cell, the averages of kappa by arm will be wide regardless. You might consider keeping those and adding a complementary item-level mixed model that treats each screened record as one observation and includes effects for training, partner type, and their interaction, with random effects for participant and item.**

 Thank you for this recommended addition to our analysis plan. We agree that with three participants per condition, arm-level kappa estimates will be imprecise and should be interpreted cautiously. Consistent with the pilot and exploratory aims of this SWAR, we have added an item-level mixed-effects analysis to explore patterns in screening decisions while accounting for clustering by participant and by item (i.e. individual study records). We clarify that this analysis is exploratory and is intended to inform the design and analytical approach of a subsequent definitive trial, rather than to support confirmatory analyses.

**In Analysis on page 9 you plan to treat confidence intervals that cross zero as evidence of equivalence. That can be misread. Suggest you avoid equivalence language or set a small, prespecified equivalence margin for kappa differences and say any tests are exploratory.**

 We agree. Any reference to evidence of equivalence has now been removed and we further underscore that any analyses are exploratory.

**Kappa can be sensitive to prevalence. Keep kappa and percent agreement, then add one robust agreement index such as PABAK. This may make the results easier to read if the include rate is low or high.**

 Thank you for this suggestion. We have revised the protocol to include reporting of the prevalence-adjusted and bias-adjusted kappa (PABAK), alongside Cohen’s kappa and percent agreement, to support interpretation of reliability given the low number of ‘includes’ during the study selection task.

**You define the partner with 4 to 6 years as moderate experience on page 6, but label the arm “experienced”. Maybe revised labelling.**

 This was an oversight on our part and we thank you for drawing this to our attention. We have now updated our figure labels to reflect ‘moderately experienced’ and likewise updated this throughout the manuscript.

**Because one person represents each partner type, partner identity is confounded with partner type. Flag this as a pilot limitation and say that the full trial will include multiple partners per level.**

 Thank you for prompting consideration of this. We have now included a note on this limitation in our conclusion section with a view to addressing it in the future trial, where feasible.

**The Procedure seems to ask participants to vote YES when unsure. Suggest adding a sentence that explains the consequence of this rule for sensitivity and specificity, so readers know you expect higher sensitivity and possibly lower specificity by design.**

 Many thanks for this suggestion. We acknowledge the importance of this caveat and have added a note to our methods to indicate the impact and trade-off between sensitivity and specificity by reducing decision options to just two, ‘YES’ or ‘NO’.

**Time is self-reported in your feasibility outcomes. If Covidence logs can provide start or finish times, say that you will also capture those as an objective check on efficiency.**

 We have queried the availability of such logs with Covidence Support and unfortunately such data is not available. Our self-reported measure was included as a means to overcome this and have access to data on time efficiency. We nonetheless acknowledge that an objective measure would strengthen the validity and reliability of this outcome.
